# Is the Use of Glyphosate in Modern Agriculture Resulting in Increased Neuropsychiatric Conditions Through Modulation of the Gut-brain-microbiome Axis?

**DOI:** 10.3389/fnut.2022.827384

**Published:** 2022-03-08

**Authors:** Jacqueline A. Barnett, Maya L. Bandy, Deanna L. Gibson

**Affiliations:** ^1^Department of Biology, The University of British Columbia, Kelowna, BC, Canada; ^2^Department of Medicine, Faculty of Medicine, The University of British Columbia, Kelowna, BC, Canada

**Keywords:** anxiety, depression, autism (ASD), agricultural practice, ecotoxicology, glyphosate, transgeneration effects

## Abstract

Environmental exposure to glyphosate and glyphosate-based herbicides has the potential to negatively influence neurodevelopment and behavior across generations indirectly through the gut-brain-microbiome axis. Potential mechanisms by which glyphosate may elicit these effects are through the disruption of the normally symbiotic relationship of the host and the gut microbiome. Given glyphosate can kill commensal members of the microbiome like *Lactobacillus* spp., Ruminococaeae and *Butyricoccus* spp., resulting in reductions in key microbial metabolites that act through the gut-brain-microbiome axis including indoles, L-glutamate and SCFAs. Glyphosate- resistant microbes in the gut have the potential to increase the production of pro-inflammatory cytokines and reactive oxygen species which may result in increased HPA activation, resulting in increased production of glucocorticoids which have implications on neurodevelopment. In addition, maternal transfer of the gut microbiome can affect immune and neurodevelopment, across generations. This perspective article weighs the evidence for chronic glyphosate exposure on the gut microbiome and the potential consequences on the gut-brain axis correlated with increased incidence of neuropsychiatric conditions.

## Sub-Toxic Glyphosate Exposure May Disrupt the Gut Microbiome and Influence Neuropsychiatric Conditions

Within the English language exists an idiom – “four-letter word.” This expression often refers to a word so profane, so scandalous, that one should only define it under this veiled description. However, the most heinous word that one can hear within agriculture contains five letters - “weeds.” The heinousness of this simple word within commercial agriculture is not without reason; weeds pose a significant threat to crops through competition for nutrients and resources. Uncontrolled weed growth would result in a loss of an estimated $43 billion annually within North America alone (1). Fortunately, modern agricultural workers have access to an arsenal of herbicides used to manage and control weeds and maximize yield. One herbicide has had a significant influence on commercial farmers.

Glyphosate was first brought to market in 1974 under the trade name Roundup®. Glyphosate was revolutionary due to its broad-spectrum mechanism of action and its flexible use pattern. The creation of glyphosate-resistant crops in the early 1990's led to massive increases in its use. By 2007, glyphosate was the most widely used herbicide in the United States, with approximately 180 million pounds applied annually in commercial agriculture and another 8 million pounds applied to home gardens (2). Over the past 30 years, these increased application rates have increased maximum residue limits for many crops, leading to higher glyphosate residues within food (3). Diet is a potent mediator of gut health and can have profound implications for systemic health and wellbeing, given that the gastrointestinal system and the gut microbiome participate in bidirectional communication with multiple systems throughout the body (4). Recently, a link between the gut, the brain and the intestinal microbiome has been discovered. This gut-brain-microbiome axis provides a mechanism by which diet can influence mental health. Alterations to the gastrointestinal microbiome have been associated with multiple neuropsychiatric and neuroinflammatory disease states, including anxiety, depression, Alzheimer's, and Parkinson's disease (5, 6).

**Figure 1:**
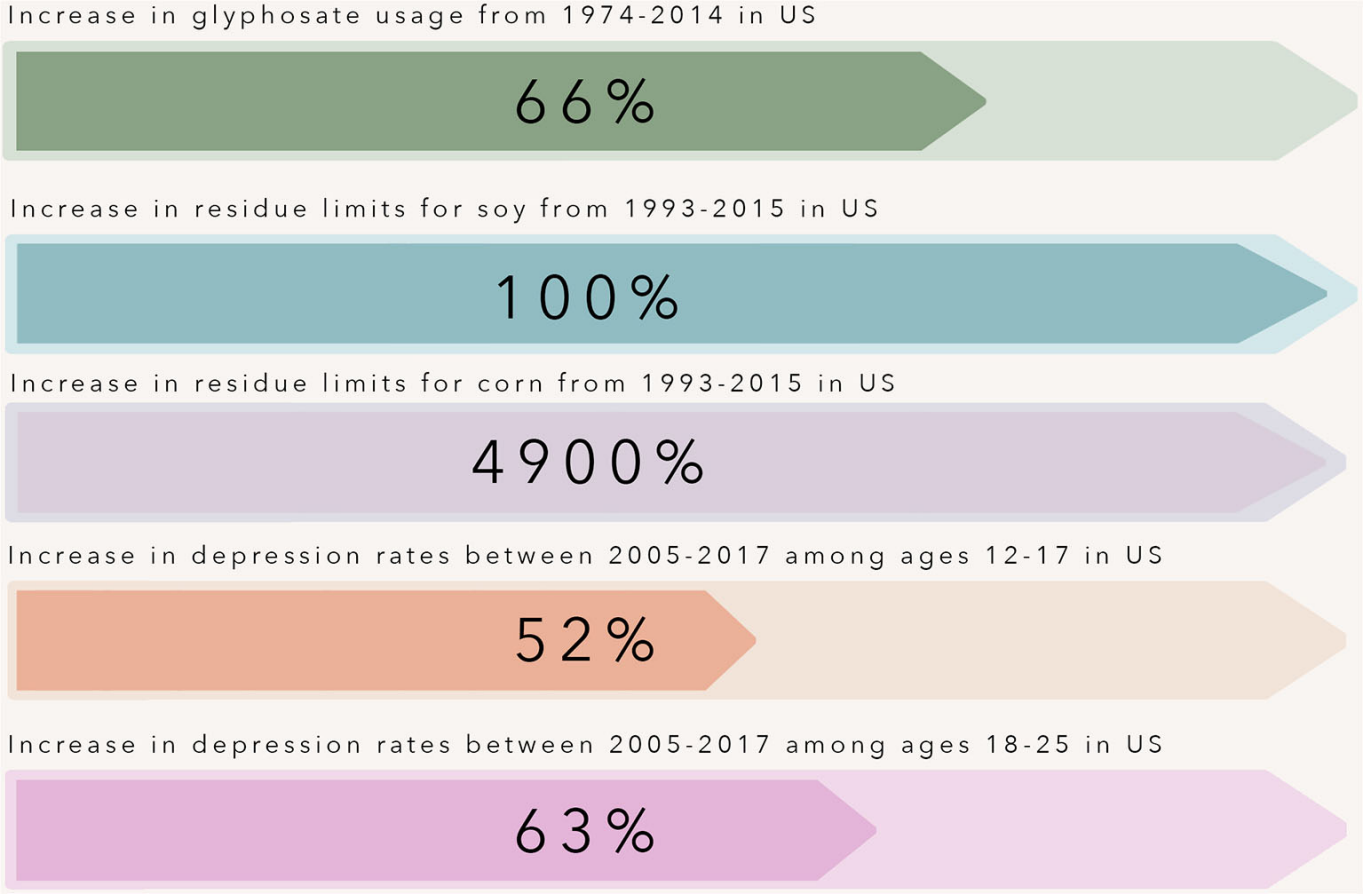
Over the past three decades, rates of depression have increased at an alarming rate in adolescents and young adults within the United States. This time point coincides with the introduction and widespread use of glyphosate-resistant corn and soy crops. The use of glyphosate-resistant crops within agriculture tripled from 1997–2009, and with this increase came increased maximum residue limits allowed for glyphosate on crops including corn and soy - crops that make up a large portion of the so-called “Western” diet pattern.

Glyphosate cornered the market with its broad-spectrum herbicidal action. However, the antimicrobial effects of glyphosate have been documented as far back as 1970. Like plants, glyphosate's toxic effects are broad-spectrum, killing many microorganisms, including bacteria, fungi (7) and protists (8). At the time, these effects would have seemed inconsequential or even beneficial for controlling plant pathogens. Now, it raises concerns regarding glyphosate's influence on human health and behavior through secondary means, such as the gastrointestinal microbiome, given what is now known regarding the gut microbiome and its influence on human health and disease. Adding to this concern is that rates of anxiety and depression among youth are increasing at an alarming rate. In North America, rates of anxiety have nearly doubled between 2008 and 2018 in those aged 18–25 (9). While these increases could be due to mental health being more openly discussed now than in the past, the period also coincides with the introduction and widespread use of glyphosate-resistant corn and soy crops. The use of glyphosate-resistant crops within agriculture tripled from 1997–2009 (10). With this came increased maximum residue limits on crops including corn and soy - crops that make up a significant portion of the so-called “Western” diet pattern (Figure 1). The acceptable daily intake (ADI) for glyphosate refers to the amount of glyphosate one can be exposed to without suffering any direct toxicity. America has one of the highest ADI's in the world, currently set at 1.75 mg glyphosate/kilogram body weight/day (11). Exposure to glyphosate below the current ADI might not directly pose a risk to human health; however, it may have consequences for health and neuropsychiatric disease susceptibility through secondary means such as the gut-brain-microbiome axis, and these effects may be felt for many generations. Here we share our perspective proposing mechanisms by which sub-toxic glyphosate exposure can influence brain development and behavior across generations and pose directions for future research. However, more in-depth and mechanistic research is needed to truly understand the causality of the relationship between glyphosate exposure and mental health.

## The Gut-Brain-Microbiome-Axis

The gastrointestinal system and its commensal microbiome participate in an intricate cross-talk with the central nervous system. Dietary components including tryptophan, glutamate and complex carbohydrates serve as a substrate for many bacterial metabolites. Amino acids, including tryptophan and glutamate, serve as precursors in synthesizing many neuroactive substances, including kynurenine, serotonin (5-HT), dopamine, gamma-aminobutyric acid (GABA) and epinephrine (12, 13). Enterochromaffin cells within the intestinal epithelium are the primary producer of gut-derived 5-HT. Tryptophan metabolites are vital precursors in the synthesis of kynurenine and 5HT within the gut. These metabolites can also enter circulation and cross the blood-brain barrier thereby influencing serotonin synthesis within the brain (14). Moreover, certain commensal microbiome members, including *Clostridium sporogenes* and *Lactobacillus* spp., can metabolize tryptophan into compounds such as 3-Indole Propionic Acid (IPA) and Indole-3-Aldehyde (I3A) (15). IPA is a potent neuroprotective antioxidant that decreases activation of glial cells and astrocytes, prevents DNA damage, and inhibits beta-amyloid fibril formation, the primary contributor to the pathogenesis of Alzheimer's disease (15). I3A acts on the aryl hydrocarbon receptor (AhR) within intestinal immune cells, resulting in increased IL-22 production, a key cytokine in maintaining mucosal homeostasis, by playing a prominent role in inflammation and tissue regeneration (15). Therefore, the gut serves as a gateway that sets off molecular mechanisms that have far-reaching consequences in the brain.

The gut microbiome normally has a symbiotic relationship with the host that shapes the immune system, particularly during early life (16). Dendritic cells routinely sample the microbes interacting with the gut epithelium and signal B and T lymphocytes to mount an appropriate immune response (16). Cytokines produced within the lamina propria, including IL-6, TNF-α and IL-1β, can enter systemic circulation and elicit wide-reaching effects. These cytokines have been shown to have potent neuroinflammatory properties. IL-6 and TNF-α are expressed by many cells within the brain, including astrocytes, microglia and neurons (17, 18). IL-6 plays a crucial role in maintaining homeostasis within neural tissue through the differentiation of oligodendrocytes and peripheral nerves' regeneration (19), whereas TNF-α results in the activation of glial cells and regulates functions including tissue remodeling (20). However, high levels of IL-6 and TNF-α have been shown to play a critical role in the pathogenesis of neuroinflammatory disorders, including multiple sclerosis (19), Parkinson's (21), and Alzheimer's disease (22). GABA is the primary inhibitory neurotransmitter within the central nervous system (CNS). Several members of the commensal microbiome, including *Bacteroides* spp. (23) and some *Lactobacillus* spp. (24, 25), have been found to synthesize GABA within the gut by using the amino acid glutamine as a precursor. Given that GABA promotes feelings of relaxation, research is being conducted into how to harness these bacteria's therapeutic potential to treat anxiety disorders and depression.

Dietary carbohydrates that are not broken down within the small intestine pass into the large intestine, where bacteria metabolize them into short-chain fatty acids (SCFA.) SCFA's play a critical role in maintaining barrier function and homeostasis, as they are the primary energy source for colonocytes (26). Moreover, SCFAs aid in the stimulation of enteroendocrine cells (L-cells), which produce gut hormones including glucagon-like-peptide 1 (GLP-1), peptide YY (PYY) (27) and cholecystokinin (CCK) (28). GLP-1, PYY and CCK enter systemic circulation where they act on the brain to promote satiation (29). These hormones also travel to the liver through the portal vein, regulate blood glucose homeostasis, and aid in protein and fat digestion. CCK is also found throughout the CNS, and high CCK levels have been shown to promote anxiety and panic disorders within humans (30) and rodents (31–33).

In addition to communication through metabolites, the gut and the brain directly communicate through the enteric nervous system. Enteric glial cells and enteric neurons found within the myenteric and submucosal plexus facilitate communication between the enteric nervous system and the CNS. Afferent neurons of the vagus nerve innervate the intestinal epithelium (34). These neurons have receptors that recognize microbial metabolites and microbial-derived neurotransmitters and neuropeptides and transmit these signals to the CNS, regulating many biological processes (35–37). This communication is bidirectional, and the CNS can elicit many changes within the gastrointestinal system. In response to stress, the CNS activates the hypothalamic-pituitary-adrenal (HPA) axis. The hypothalamus begins producing corticotropin release factor, prompting the pituitary to release adrenocorticotropic hormone, initiating the synthesis and release of cortisol from the adrenal glands. Cortisol prepares the body to deal with a stressor through the modulation of several systems. Cortisol triggers the breakdown of glucose and glucagon inhibition, thereby raising blood glucose levels, providing the body with enough energy to fight or flee from the stressor. Cortisol also inhibits digestion and influences intestinal barrier integrity. The presence of microbes in the gastrointestinal tract contributes to the development of the HPA axis and the stress response in infants. Fecal microbiota transplantation from specific-pathogen free (SPF) mice to recipient germ-free mice resulted in some recovery of the altered HPA axis response in mice born germ-free, but only if the transplant was performed at an early stage of life, suggesting the role of a colonized microbiome for the development of the HPA axis (38).

**Figure 2:**
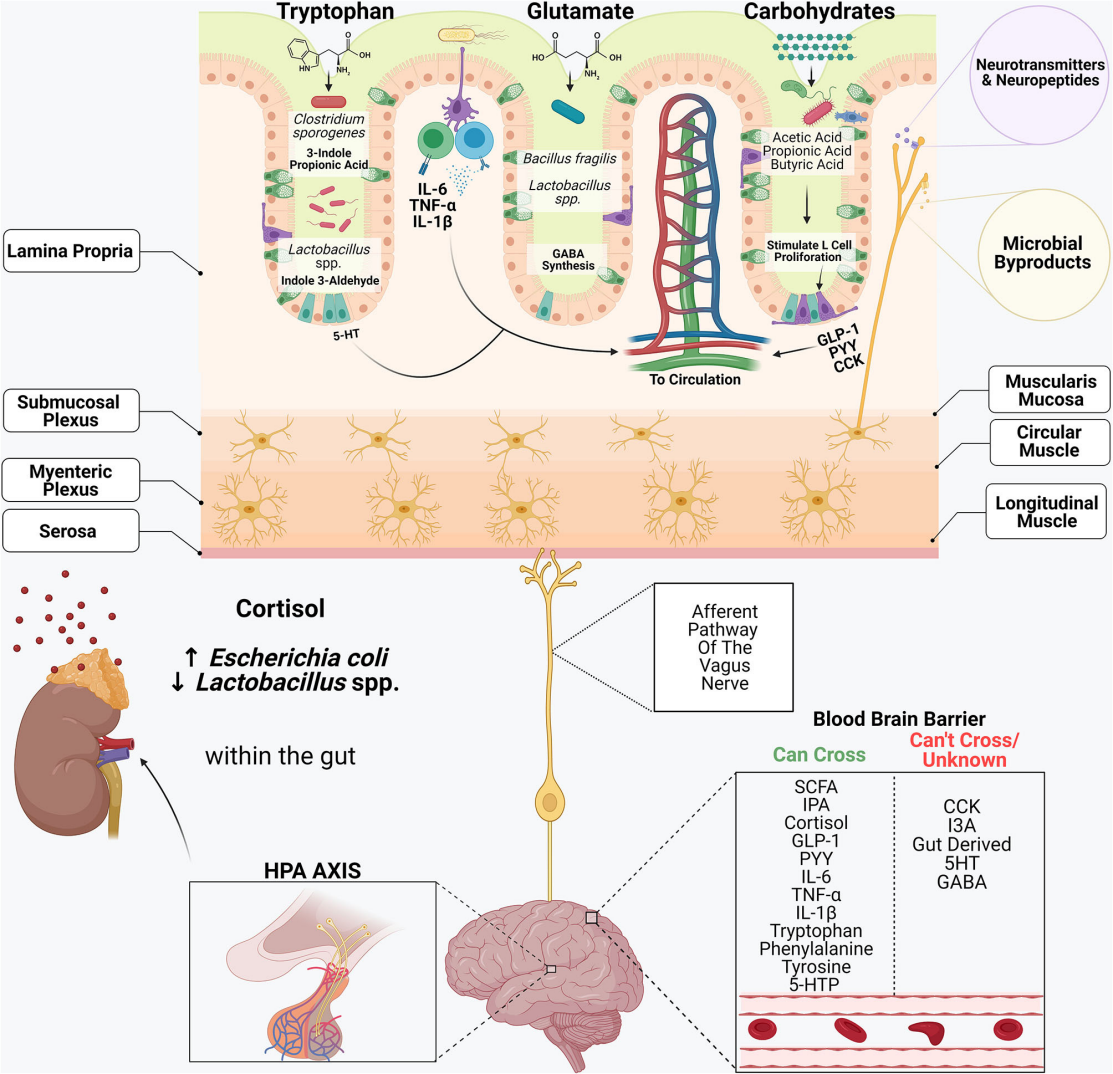
The gastrointestinal system and its commensal microbiome participate in an intricate cross-talk with the central nervous system. Dietary components including tryptophan, glutamate and complex carbohydrates serve as substrates for many bacterial metabolites. Amino acids, including tryptophan and glutamate, serve as precursors in synthesizing many neuroactive substances, including serotonin (5-HT), dopamine, gamma-aminobutyric acid (GABA) and epinephrine. Enterochromaffin cells within the intestinal epithelium are the primary producers of gut-derived 5-HT. Certain commensal microbiome members, including *Clostridium sporogenes* and *Lactobacillus* spp., metabolize tryptophan into compounds such as 3-Indole Propionic Acid (IPA) and Indole-3-Aldehyde (I3A.) Cytokines produced within the lamina propria, including IL-6, TNF-α and IL-1β, can enter systemic circulation and elicit wide-reaching effects. *Bacteroides* spp. and some *Lactobacillus* spp., synthesize GABA within the gut by using the amino acid glutamine as a precursor. Short-chain fatty acids play a critical role in the stimulation of enteroendocrine cells (L-cells), which produce gut hormones including glucagon-like-peptide 1 (GLP-1), peptide YY (PYY) and cholecystokinin (CCK.) Afferent neurons of the vagus nerve innervate the intestinal epithelium and contain receptors that recognize microbial metabolites and microbial-derived neurotransmitters and neuropeptides and transmit these signals to the CNS, regulating many biological processes. In response to stress, the CNS activates the hypothalamic-pituitary-adrenal (HPA) axis. Cortisol also inhibits digestion and influences intestinal barrier integrity, and alters the composition of the microbiome.

These findings highlight the delicate balance between diet, the gastrointestinal microbiome and behavior. Dietary components including amino acids, fiber and fats are potent mediators of the gastrointestinal microbiome and its metabolites. Microbial metabolites, including tryptophan, SCFA and bile acids, can elicit effects on the CNS and metabolism. Simultaneously, prolonged stress and activation of the HPA axis influences intestinal permeability and can alter the gastrointestinal microbiome's composition, promoting the growth of opportunistic pathogens and decreasing genera of bacteria considered beneficial (Figure 2). It is clear that the gut and brain are interconnected, and both can influence one another. What remains unclear and of concern is how environmental toxins, including glyphosate, affect the type of microbes present in the gut and, therefore, alter the gut-brain axis.

### Glyphosate Changes Gut Microbes to Both Provoke and Promote Neuropsychiatric Conditions

Glyphosate works by inhibiting the Shikimate pathway, a metabolic pathway involved in synthesizing aromatic amino acids in both plants and microorganisms. In animal models, both glyphosate and glyphosate-based herbicides have elicited changes to the gut microbiome composition. While some taxonomic changes appear to be either dose-dependent or formulation-dependent, specific taxa are particularly susceptible to glyphosate's bactericidal effects (39). One such taxon is *Lactobacillus* spp., a minor constituent of the human gastrointestinal microbiome which plays a significant role within the gut-brain-microbiome axis. *Lactobacillus* spp., can break down tryptophan within the gut into indoles, an essential mediator in the gut-brain axis (40). As described above, I3A, aids in maintaining mucosal barrier function through AhR resulting in IL-22 production (41). AhRs mediate many pro and anti-inflammatory pathways throughout the body and have been suggested as a mediator in the relationship between environment, diet, microbes and host response (42). The interaction between bacterially derived indoles and AhR has been demonstrated to confer a wide range of health benefits ranging from extended healthspan (43) to reductions in pro-inflammatory cytokines involved in the pathogenesis of brain amyloidosis and cognitive decline in the elderly (44).

Both glyphosate and glyphosate-based herbicide exposure have been shown to decrease members of the family Ruminococcaceae (45). Reductions in Ruminococcaceae within the gut microbiome are implicated in multiple neuropsychiatric disease states, including Parkinson's disease (46), Schizophrenia (47), depression (48) and modulate social behaviors in rodents (49). Members of the family Ruminococcaceae produce a variety of metabolites that can alter mood and behavior, including L-glutamate. The amino acid L-glutamate mediates communication between the gut and the brain in several ways. First, L-glutamate interacts with receptors present within the luminal side of the gut and transmits information to adjacent cells and neurons. L-glutamate sensing mechanisms are located on both afferent and efferent fibers of the abdominal vagus nerve (50). L-glutamate is also a precursor in synthesizing GABA providing a potential mechanism for how gut bacterial metabolites affect mental health since reductions in GABA within the CNS are associated with neuropsychiatric disease states, including depression (51), anxiety (52) and premenstrual dysphoric disorder (53). Reductions in GABA-producing bacteria within the fecal microbiome are negatively correlated with brain signatures associated with depression (13). Other members of the gastrointestinal microbiome which play a essential role in the synthesis of GABA include *Bacteroides* spp., and *Lactobacillus* spp., both of which are highly susceptible to glyphosate (54). While it is unclear if gut-derived GABA can cross the blood-brain barrier and directly influence levels within the CNS, administration of GABA producing bacteria have been shown to influence neurotransmitter receptor expression within the CNS of healthy animals, including reduced expression of GABA receptors within the prefrontal cortex and amygdala, two critical brain-regions in the etiology of depression and anxiety (24). Indeed, animals treated with GABA-producing bacteria, including *L. rhamnosus*, exhibited decreased anxiety and depression-like behavior, as well as decreases in stress-induced corticosterone levels (24). Maintaining the balance between excitation and inhibition within the GABAergic system is crucial for proper functioning, and early-life perturbations have been associated with the development of life-long cognitive and emotional impairments, including autism (55), schizophrenia (56), and Tourette syndrome (57).

Stress, whether it be derived from a “flight-or-fight” response or an inflammatory response, result in the same outcome within the CNS. At the first sign of trouble, the HPA axis becomes activated and releases glucocorticoids to aid in the “flight-or-fight” response. Classic “pro-inflammatory” cytokines including IL-1β, TNF- α and IL-6 can activate the HPA axis. Each of these cytokines is increased in response to high-dose glyphosate administration (58). These glucocorticoids, including cortisol, can cross the placenta and play a key role in the development and maturation of the fetal HPA axis leading to increased stress responses within offspring (59–61). Certain bacterial species including *Bifidobacterium infantis*, can have beneficial effects on abhorrent stress responses in rodents (38). Recent studies have examined the ability of certain microbes to resist the deleterious effects of glyphosate due to the presence of glyphosate-sensitive enzymes (EPSPS Class I) and glyphosate-resistant enzymes (EPSPS Class II) (39). Several subspecies of the genus *Bifidobacterium* possess the glyphosate-sensitive Class I EPSPS enzymes, suggesting *Bifidobacterium* spp., may be particularly sensitive to glyphosate's effects (39).

Exposure to glyphosate reduces the abundance of SCFA producing bacteria, including Ruminococcaceae, *Butyricoccus* spp., *Lactobacillus* spp., *Clostridium* spp., and *Bacteroides* spp. Recent studies have highlighted that in addition to maintaining gut homeostasis and barrier function, SCFAs play a fundamental role in communication within the gut-brain-microbiome axis. Exposure to non-colonized germ-free mice display altered microglial development, which is ameliorated through oral administration of acetate, propionate, and butyrate (62). Microglial cells are one of the primary immune cells found within the CNS and play a key role in promoting and resolving neuroinflammation. The role of microglia in the pathogenesis of neuroinflammatory disorders, including Alzheimer's disease and Parkinson's disease, has been extensively studied. However, emerging evidence has suggested that immune dysregulation also contributes to the pathology of mental and behavioral disorders, including obsessive-compulsive disorder (63). SCFAs play a crucial role in regulating expression levels of enzymes responsible for the formation of neurotransmitters within the CNS, including serotonin, dopamine, noradrenaline and adrenaline (62). Alterations in SCFA expression are associated with numerous disease states, including autism spectrum disorders, mood disorders including depression and anxiety, Alzheimer's disease and Parkinson's disease. Additionally, reductions in SCFA producing bacteria including *Lactobacillus* spp., and other butyrate-producing bacteria contribute to oxidative stress *via* the formation of reactive oxygen species (ROS). The CNS is particularly sensitive to the effects of ROS due to its high oxygen requirement (64). Both high dose (65) and low-dose (66) glyphosate exposure reduce key enzymes necessary for reducing ROS including superoxide dismutase and glutathione reductase. Therefore, the detrimental effects of glyphosate on the CNS may be twofold: a direct reduction in enzymes responsible for the reduction of ROS combined with a reduction in commensal microbes, which offer protective benefits in the face of damaging oxidative stress. In a similar manner, glyphosate may activate the HPA through the overexpression of pro-inflammatory cytokines, while simultaneously eliminating members of the microbiome important in alleviating stress.

Certain members of the gastrointestinal microbiome may have the ability to transform glyphosate within the gut. In the environment, the soil microbiome plays a prominent role in the breakdown of glyphosate. Approximately 70% of glyphosate is broken down into aminomethylphosphonic acid (AMPA), a molecule with similar toxicity to glyphosate. Some species found within the soil microbiome and the gastrointestinal microbiome, including *Pseudomonas* spp., and *Alcaligenes* spp., can metabolize glyphosate into sarcosine (67). Sarcosine is a naturally occurring by-product of glycine synthesis and degradation and is found in many foods. Recent studies have shown that sarcosine has neuromodulatory properties. Specifically, supplementation with sarcosine in addition to treatment with certain antipsychotics results in significant decreases in symptoms within schizophrenic patients (68). It is thought that these effects are due to sarcosine's ability to act as a type 1 glycine transporter inhibitor and glycine agonist, thereby increasing glycine concentrations within the brain and N-methyl-D-aspartate (NMDA) receptor activation (68). Treatment with sarcosine also improved major depressive symptoms within schizophrenic patients (69). However, studies have shown that stress-induced activation of NMDA receptors in otherwise healthy individuals results in microglial activation and increased pro-inflammatory responses within the CNS (70). Overall, certain members of the gut microbiome may transform glyphosate into sarcosine in addition to AMPA. While the effects of increased NMDA activation in individuals may benefit those suffering from neuropsychiatric conditions, including schizophrenia, the consequences for otherwise healthy individuals may be detrimental.

Overall, these findings suggest an intriguing correlation between glyphosate exposure, changes to the gut microbiome and abhorrent development of the GABAergic system, HPA-axis and the formation of microglial cells. Additionally, metabolism of glyphosate by the microbiome may alter NMDA receptor activation thereby promoting neuroinflammation in otherwise healthy individuals. Glyphosate-induced perturbations to the gastrointestinal microbiome may influence the synthesis of many neurotransmitters, necessary for mental health and wellbeing, encourage the formation of damaging ROS and reduce bacteria thought to be beneficial during times of stress. While the understanding of this correlation is still emerging, more research to prove or disprove this relationship is warranted given the food system is known to contain glyphosate residues.

## To Ban or Not to Ban? That is the Question

In its own right, glyphosate has become a scandalous, profane, “four-letter word.” A word like politics and religion, not to be discussed at the dinner table for fear of inciting debate. It is appealing to villainize glyphosate; however, the fact remains that it is a valuable tool that does convey some authentic benefits to society that often go unnoticed. The use of glyphosate and glyphosate-resistant crops has allowed for less greenhouse gas emissions from reduced tractor usage, fewer spray runs, and reduced tillage, and these practices have resulted in increased carbon retention within the soil. It is estimated that a widespread ban on glyphosate would increase carbon emissions, analogous to putting 11.77 million cars on the road (71). However, the fact remains that glyphosate may also pose a genuine risk to human health, development and disease susceptibility, and these consequences may be felt for many generations, given that recent studies have shown that glyphosate and glyphosate-based herbicides can persist within the environment for years or even decades following application (72).

The study of glyphosate itself poses many unique challenges given that it is a small, non-volatile molecule that is easily ionized. These challenges make analyzing food samples difficult and inaccurate, resulting in arbitrary maximum residue limits being set for crops and a distinct lack of routine monitoring for glyphosate levels within our food supply. Additional steps to increase recoveries such as derivatization with reverse phase chromatography and extraction optimization are time-consuming, costly, and rarely performed in routine, large scale surveillance. This lack of transparency combined with the non-physiologically relevant doses used in much of the research conducted to date has perpetuated a cycle of fear among the public.

Here we highlight several ways glyphosate can influence mental health and behavior through changes in the gut-brain-microbiome axis. However, the doses used within the literature to support these theories are much higher than what the average North American would be exposed to through diet or environmental exposures alone. Ultimately, better detection methods and routine monitoring are needed to determine the levels of glyphosate people are exposed to daily. Then, more research needs to be done utilizing these physiologically relevant doses and the impact on human health to help guide glyphosate usage and maximum residue limits moving forward. Future long-term multi-generational studies examining the consequences of low-dose glyphosate exposure throughout several generations are needed to understand better the long-term implications of glyphosate use, given its pervasiveness in the environment. It is entirely possible that glyphosate may be the next DDT – where it may continue to elicit its detrimental effects long after its use is stopped. Moreover, future studies investigating diet patterns, including the ketogenic, vegetarian and gluten-free diets, would be invaluable given how much misinformation exists within the public domain regarding these diet patterns and their potential for reduced dietary glyphosate exposure with no scientific evidence to back up such claims (73, 74). Additionally, more transparency is needed regarding the composition of proprietary glyphosate-based herbicides to determine the effects of chronic exposure to these adjuvants on health. Overall, we posit that environmental exposure to glyphosate and glyphosate-based herbicides has the potential to negatively influence neurodevelopment and behavior across generations.

## Data Availability Statement

The original contributions presented in the study are included in the article/supplementary material, further inquiries can be directed to the corresponding author.

## Author Contributions

JB and MB critically reviewed and summarized all literature, JB drafted the figures and the manuscript. DG conceived of the study with insights from JB and MB, provided oversight, supervised the project, and critically evaluated the manuscript. JB, MB, and DG edited the manuscript and approved the final version. All authors contributed to the article and approved the submitted version.

## Funding

JB is funded by a PGS-D from the Natural Sciences and Engineering Research Council (NSERC.) MB is funded by a UBC Undergraduate Research Award (URA.) DG has no funding sources that were used in this study. Any glyphosate research conducted in the Gibson lab has been funded by UBC crowd funding: https://crowdfundraising.ubc.ca/projects/what-in-the-wheat/.

## Conflict of Interest

The authors declare that the research was conducted in the absence of any commercial or financial relationships that could be construed as a potential conflict of interest.

## Publisher's Note

All claims expressed in this article are solely those of the authors and do not necessarily represent those of their affiliated organizations, or those of the publisher, the editors and the reviewers. Any product that may be evaluated in this article, or claim that may be made by its manufacturer, is not guaranteed or endorsed by the publisher.
